# An In Vitro Study Comparing the Antibacterial and Mechanical Properties of Zinc Oxide-Based Nanofillers in Orthodontic Adhesives for White Spot Lesion Prevention in Fixed Orthodontic Therapy

**DOI:** 10.7759/cureus.66967

**Published:** 2024-08-15

**Authors:** Fatima Saeed, Muhammad Ilyas, Asmi Shaheen

**Affiliations:** 1 Department of Orthodontics, de' Montmorency College of Dentistry, Lahore, PAK

**Keywords:** ari, enamel demineralization, material characterization, preventive dentistry, sem, novel orthodontic adhesive, sbs, transbond xt, orthodontic bonding, white spot lesion

## Abstract

Introduction

Preventing enamel demineralization (white spot lesions or WSLs) around the brackets during and after orthodontic therapy has been a challenging problem. Zinc oxide (ZnO) nanoparticles (NPs) show antibacterial effects against cariogenic bacteria Streptococcus (S.) mutans.

Materials and methods

In this study, researchers modified Transbond XT adhesive (Sigma Aldrich, St. Louis, Missouri, USA) by adding different concentrations of ZnO nanoparticles, i.e., 0.1% and 0.5wt%, in two experimental groups and a control group. We performed Fourier transform infrared spectroscopy and scanning electron microscopy for physio-structural characterization and investigated antibacterial ability by disc diffusion and colony-forming tests. We conducted shear bond strength and adhesive remnant index to determine the mechanical characteristics.

Results

The development and size of the inhibition zone were greatly dependent on the concentration of ZnO nanoparticles in the disc agar diffusion test. All ZnO NP-based experimental adhesives reduced the colony numbers for S. mutans. For S. mutans, the composite comprising 0.5wt% ZnO nanoparticles significantly reduced colony counts. The control group exhibited the maximum mean shear bond strength, whereas 0.5wt% nanoparticles composite had the lowest number.

Conclusion

Adding ZnO as nanofillers imparts antibacterial properties to the orthodontic adhesives. An increase in the concentration of ZnO nanoparticles in orthodontic adhesive increases its antibacterial properties. We found the shear bond strength of the novel composite with up to 0.5wt% ZnO nanoparticles to be in a clinically acceptable range.

## Introduction

In routine orthodontic practice, brackets are attached to the tooth surface with the help of a bonding technique using resin-based cement, that is, composite. Despite having several advantages, such as simplicity of use and superior esthetics, this technique that uses dental composite as the adhesive has some drawbacks, including white spot formation, poor plaque control, and bond failure.

White spots are the most common and visually unpleasant of all shortcomings. In the vicinity of the bracket and adhesive, WSLs are brought on by bacterial acid produced by plaque biofilm bacteria. WSLs may develop around brackets within a month of bracket introduction, as opposed to conventional carious lesions, which typically take at least six months to manifest. Cured resin composites often lack antibacterial activity, causing bacterial adhesion and plaque formation on their surfaces. Adopting an appropriate preventive strategy is extremely important, given how quickly WSLs can develop and become irreversible. Modern dentistry is focused on a preventive approach rather than invasive restorations of carious teeth [1-4].

Nanotechnology can be described as the exploration, synthesis, and characterization of materials ranging from 1 nm to 100 nm. Hence, several studies have attempted to evaluate the mechanical and antibacterial characteristics of numerous nanoparticles integrated into orthodontic adhesives [5-6]. Since enamel demineralization occurs at the nano level, incorporating nanoparticles with antibacterial and anti-inflammatory potential in orthodontic adhesives to prevent WSLs is logical [6-8]. The intrinsic characteristics of metal nanoparticles, for instance, zinc (Zn), silver (Ag), and titanium (Ti) are determined mainly by their composition, size, crystallinity, shape, and morphology [9].

Dentistry's most frequently used nanoparticles (NPs) comprise various materials, including carbon, lipids, hydrogel, silica, hydroxyapatite, polymers, dendrimers, and metals or metal oxides [9]. The enhanced bactericidal activity of NPs with antibacterial properties is due to the electrostatic interaction of positively charged NPs with the bacterial cell wall upon contact and their concurrent targeting of several biomolecules. NPs’ small dimensions enhance their mechanical capabilities and antibacterial effectiveness while minimizing adverse effects, including hypersensitivity or allergic reactions [10]. It is possible to lessen the formation of biofilms by using metal oxide NPs such as ZrO_2_, ZnO, TiO_2_, and MgO [11].

ZnO has recently emerged as an alternative to other metal oxide nanoparticles because it is theoretically known to be biocompatible, nontoxic, and does not cause discoloration [12]. ZnO has antibacterial effects against various bacteria and fungi. The ZnO NPs’ small diameter, size, and increased surface-to-volume ratio contribute to their enhanced antibacterial activity [13].

Incorporating antibacterial agents as nanofillers in orthodontic bonding adhesives has been known to affect their mechanical properties such as shear bond strength (SBS), tensile strength, and polymerization shrinkage. Some researchers have reported decreased mechanical properties of adhesives with increasing concentrations of antibacterial agents in orthodontic bonding adhesives. Some studies suggest that incorporating antibacterial agents does not affect the SBS of orthodontic adhesives [14-15].

Considering the distinctive features of ZnO NPs, the present study targets the analysis and comparison of antibacterial properties and mechanical characteristics, such as shear bond strength (SBS) and adhesive remnant index (ARI), of ZnO NP-based orthodontic adhesive (Transbond XT; (Sigma Aldrich, St. Louis, Missouri, USA) in two concentrations (0.1% and 0.5%) to prevent white spot lesions (WSLs) around the brackets in fixed orthodontic intervention.

## Materials and methods

This comparative in vitro study was conducted after obtaining ethics approval vide IRB number 1867 dated 10-08-2023 from the institutional review board of de Montmorency College of Dentistry, Lahore. This study used healthy maxillary first premolars extracted for orthodontic purposes. Before we used their extracted teeth, patients gave informed consent. Healthy maxillary first premolars were acquired from orthodontic patients ranging from 15 to 30 years old according to the following inclusion and exclusion criteria. Inclusion criteria were vital, non-carious, and healthy maxillary first premolars. Teeth with enamel pathologies like fluorosis or hypoplasia that have undergone endodontic therapy or restorative procedures for traumatic non-vital teeth were not considered for this study.

ZnO nanoparticles preparation

Synthetic ZnO dispersion NPs (20 wt% in water, <100 nm in size transmission electron microscopy (TEM)) were purchased from Sigma Aldrich Chemical Company. We poured the ZnO dispersion into a Petri plate (15 ml), froze it in liquid nitrogen, and freeze-dried it using a vacuum freeze dryer (Ilshin Lab. Co. Ltd., Seoul, Republic of Korea) at a pressure of 26.5 Pa. All the moisture was sublimed from the frozen product by freeze drying, and dried particles were obtained after lyophilization [16].

Preparation of experimental adhesive specimens

Transbond XT adhesive was plasticized with 99% ethanol and added dropwise. Next, we carefully mixed 0.1 wt% and 0.5 wt% of experimental powders (ZnO) until a homogeneous mixture is achieved [16]. This procedure was completed in a dimly lit room with a humidity level of 55% and an ambient temperature of 23 °C ± 3 °C. The final adhesives were stored in sealed vials and wrapped with aluminum foil to prevent premature polymerization. Carefully chosen teeth were randomly assigned to two experimental and control groups. Every group contained 14 teeth. This study has one control group (Transbond XT) and two experimental groups, 0.1 wt% and 0.5 wt% ZnO-based orthodontic adhesives.

Characterization of experimental adhesives

FTIR

A Fourier-transform infrared spectrophotometer (FTIR) (Nicolet 6700, Thermo Fisher Scientific, Waltham, Massachusetts, USA, was used for functional group analysis over the region of 650-4000 cm-1, with a resolution of 8 cm-1, averaging about 128 scans. We employed the FTIR spectroscopic analysis using the device’s software to first analyze the functional groups in experimental adhesives in absorbance mode.

SEM

A scanning electron microscope (SEM) (Vega LMU TESCAN, Brno, Czech Republic) measured the topography and surface morphology of the novel materials at 10,000X magnification. It produced images using SEM with a focused electron beam directed at the specimen’s surface.

Preparation of composite discs

In this investigation, composite discs (n = 14) were prepared for each study group. The control group consisted solely of Transbond XT composite discs. The orthodontic adhesive was packed in circular metal molds with a 7 mm diameter and supported by glass slides on both sides to produce specimens. We removed the composite discs from the molds after light curing for 20 seconds on each side and then sterilization [15].

Disc diffusion agar method

The disc diffusion test was carried out to investigate the antibacterial activity of ZnO composites using the American Type Culture Collection (ATCC 700610, Manassas, VA) S. mutans. From an S. mutans agar plate culture, we extracted three to five colonies of the same morphological type and transferred them using a sterile loop into a tube containing 9 ml of brain heart infusion (BHI) broth. The tube was then incubated overnight at 37 °C. Afterward, the optical density (OD) was set to 0.2 while keeping the absorbance wavelength at 600 nm by using the UV-Vis DRS spectrophotometer Lambda 35. At the same time, agar plates were inoculated with 200 µl of bacterial BHI broth with S. mutans (0.2 OD). Using a sterile L spreader, 200 ml of the broth was transferred to the center of a dry agar and evenly distributed over the entire agar surface. Seven mm diameter discs of experimental adhesives were sterilized (n = 14) by UV in a fume hood and then immediately placed over the plates. The agar plates were incubated with experimental discs for 48 hours at 37 °C. Following the incubation period, the plates were examined for uniform culture growth (biofilm production) and the development of inhibitory zones around the discs, which were measured using a Vernier caliper in millimeters. The diameter of each inhibitory zone for each disc was measured three times, computing the mean of those values. The center of the disc was considered zero and measured the distance from the center to the edge of the disc with zero bacterial growth [16]. Statistical significance between the study groups was found as a result of statistical analysis (i.e., one-way analysis of variance (ANOVA)) by keeping the p-value at 0.05.

Colony-forming test or dilution test

A quantitative analysis of the specimens’ bactericidal abilities against the carcinogenic S. mutans bacteria was conducted. The nutrient broth was inoculated with a single bacterial colony and then grown at 37 °C overnight in an autoclave. Colony-forming units (CFUs) on agar plates were used to measure the impact of the experimental substance on bacterial growth after 24 hours. The agar plates with sterilized samples were incubated for 48 hours at 37 °C. The plates were infected with 350 μl of 1 × 10^6^ CFU/ml of each bacterium suspension. We identified and counted the colonies (CFU/ml) [17].

Shear bond strength testing

Seventy healthy human maxillary first premolars extracted for orthodontic reasons were used for SBS and ARI tests according to the ISO 29, 022:2013 guidelines. After cleaning and debridement, we randomly allocated them to three study groups (n = 14). After that, a 37% phosphoric acid gel (3M Unitek Orthodontic Products, Monrovia, California, USA) was applied for 30 seconds before rinsing and gently air-drying them for another 30 seconds. The base of the stainless-steel orthodontic brackets (standard edgewise, 0.022-in slot, 12.62 mm^2^ bracket concave base area) was coated uniformly with adhesive (3M Unitek), the brackets were placed on the labial/buccal surface of the teeth, and then exposed to curing light (Woodpecker, London, UK) for 20 seconds on each side. Orthodontic metal brackets of maxillary first premolars were considered to bond experimental samples. The teeth bonded with brackets were placed in a 37 °C water bath overnight before being put through thermocycling per ISO/TS 11405:2015 guidelines. We performed thermocycling on each specimen for 500-1000 rounds a day to re-create temperature fluctuations in the mouth cavity. Each cycle included a 15-second immersion in a water bath maintained at a temperature of 5 °C, a 10-second dwell period, and a 15-second immersion in a 55 °C water bath. After thermocycling, the teeth were secured to the corners of round metal molds (2.5 cm diameter) using rectangular wire. The mold was then filled with self-curing acrylic resin (Acropars, Iran) up to the cementoenamel junction level. The bracket base of the specimens was placed parallel to the direction of the applied force. The composite-bracket interface was sheared using a 0.6-mm metal blade in an incisogingival or occlusogingival direction at a crosshead speed of 1.0 ± 0.1 mm/min. The buccal surface of the tooth was positioned parallel to the chisel to ensure the blade of the universal testing machine made contact with each bracket from the incisal aspect as close to the bonding interface as possible. The Roell-7060 universal testing machine (Zwick/Roell, Ulm, Germany) was employed to measure the SBS. The most significant force needed to separate a bracket was determined and the mean SBS (N) for each group. The SBS was calculated in megapascals (MPa) by dividing the acquired value (N) by the bracket surface area (mm^2^) [17].

Adhesive remnant index

Evidence of residual adhesive adhering to the bracket base was assessed using a stereomicroscope at 10X magnification (SMZ800, Nikon, Tokyo, Japan) after bracket debonding. Adhesive remnants were scored as per the ARI given in Table 1 [16]. Each tooth’s residual resin was quantified as a percentage of the resin still present around the bracket’s edge [15-17].

**Table 1:** Adhesive remnant index Source: [16]

Scores	Adhesive Remnant Index
0	No or negligible adhesive on bracket
1	≤ 25% adhesive on bracket
2	Adhesive ranging 25–50% on bracket
3	Adhesive ranging 50–75% on bracket
4	Adhesive ranging 75–100% on bracket

Statistical analysis

The obtained data was recorded in tabulated form and then entered into SPSS software version 26 (IBM Corp., Armonk, NY, USA). Descriptive data were obtained for frequency, percentage, and mean standard deviation. The ANOVA analysis was employed to ascertain differences between the study groups. The chi-square test was employed to analyze ARI scoring, which scores we inferred to be significant at p-values less than 0.05.

## Results

Characterization

FTIR Spectroscopic Analysis

**Figure 1:**
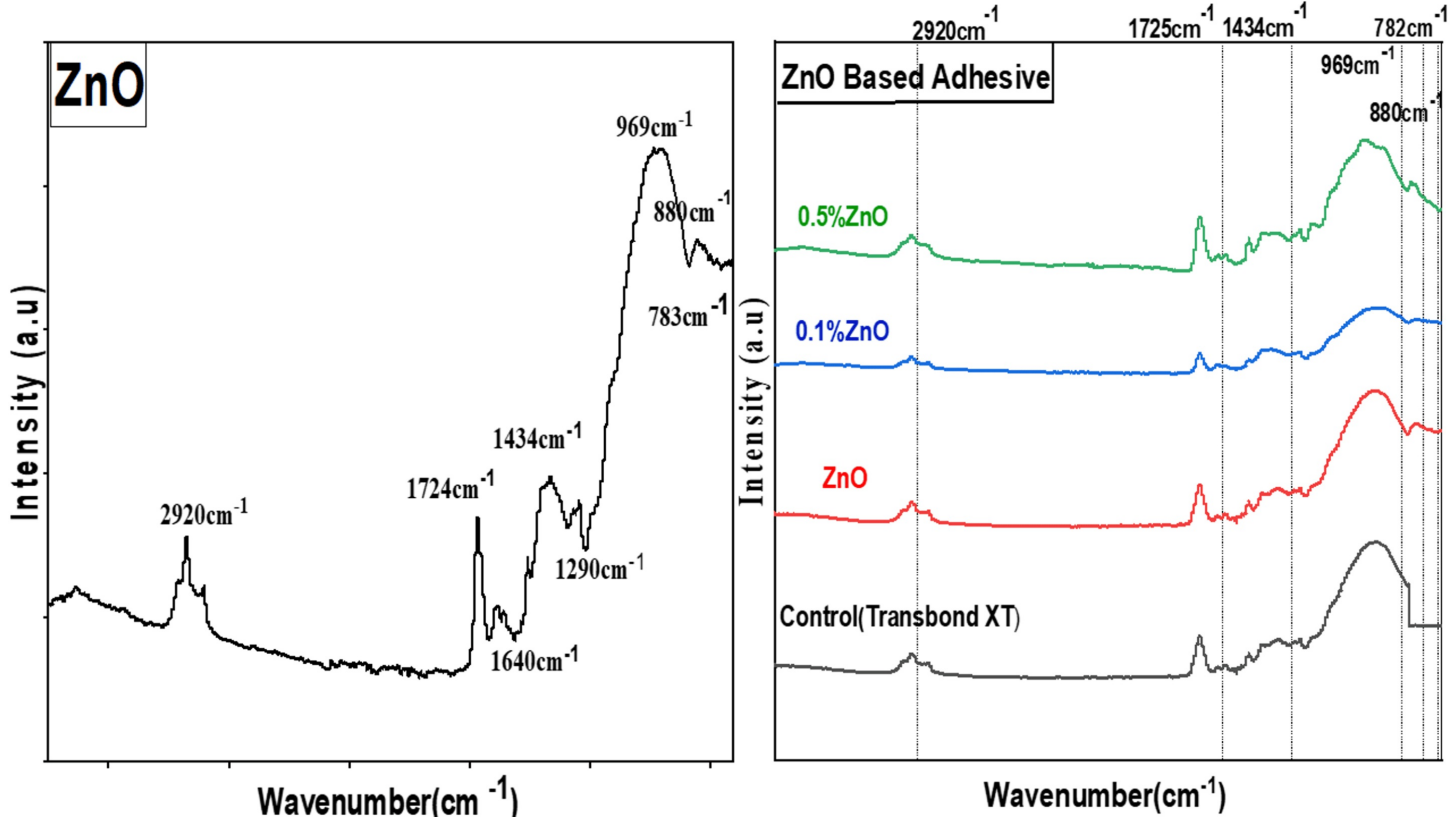
FTIR of ZnO nanoparticles and ZnO-based adhesives FTIR: Fourier transform infrared spectroscopy

ZnO NP FTIR typically exhibits peaks at 621 cm-1 and 786 cm-1. ZnO NPs’ FTIR spectra, measured between 650 and 5000, show that the particles had bands at 2920 cm-1, 1724 cm-1, 1643 cm-1, 1434 cm-1, 1288 cm-1, 1194 cm-1, 969 cm-1, 880 cm-1, 782 cm-1, and 682 cm-1. Figure 1 shows the results of the FTIR examination of the synthesized NPs, which demonstrated the presence of the ZnO functional group at a low wave number, which was compatible with the results of numerous investigations. The peaks of ZnO NPs appear in the FTIR spectra of ZnO-based adhesives (Figure 1) at 1724 cm-1, 969 cm-1, 1434 cm-1, 880 cm-1, and 782 cm-1, confirming the presence of ZnO in the experimental adhesive [16-18].

**Figure 2:**
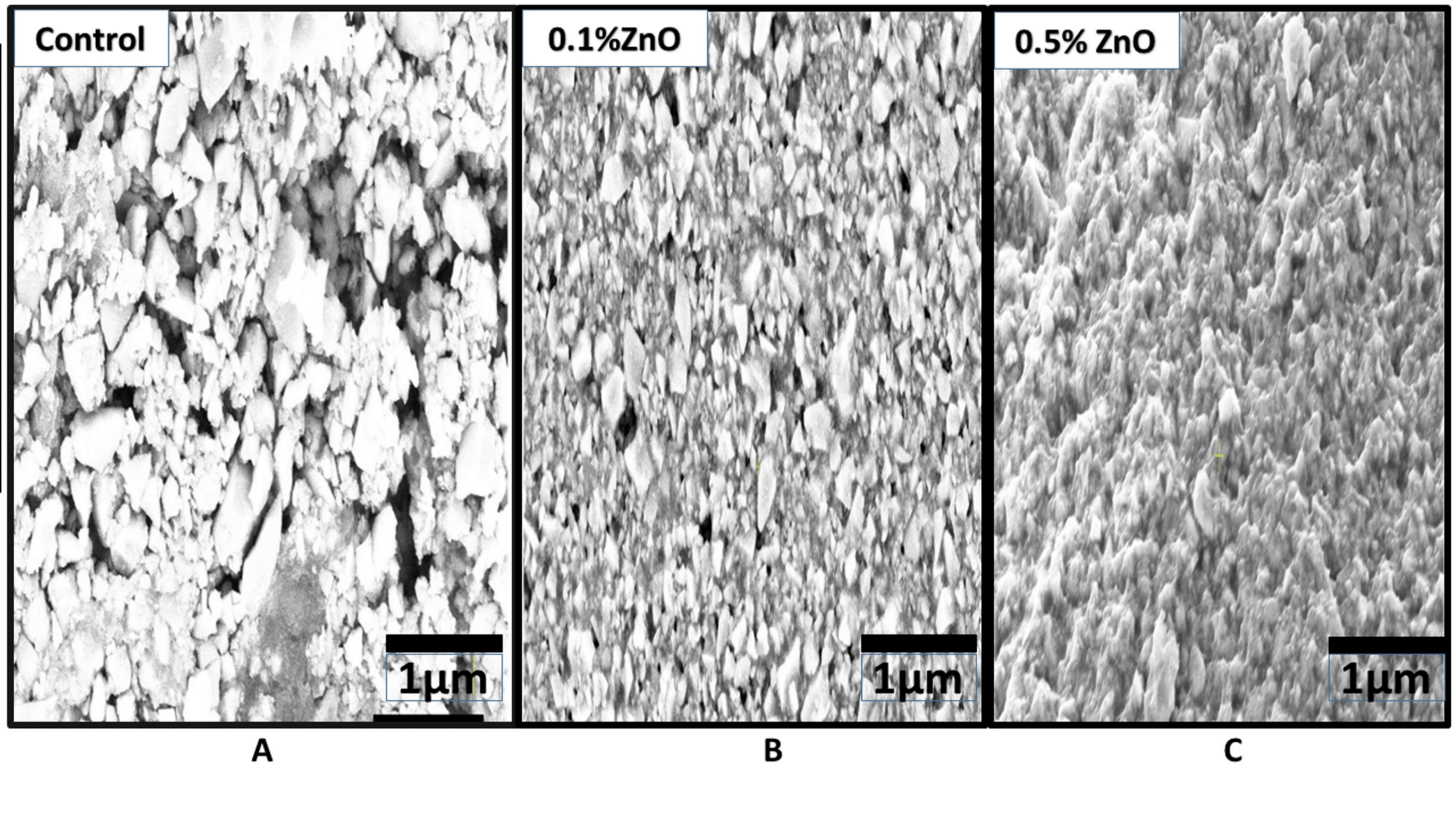
SEM of the study groups: A - Control (Transbond XT), B - 0.1% ZnO-based orthodontic adhesive, and C - 0.5%ZnO-based orthodontic adhesive SEM: scanning electron microscopy

The SEM analysis shows agglomeration of different shapes and sizes of the ZnO NPs in ZnO-based adhesives (Figure 2), and ZnO NPs have a size of less than 1 μm, ranging from 0.21-0.36 μm in size. Experimental adhesives incorporating 0.5% and 1.0% ZnO NPs revealed the homogenous distribution of irregularly shaped ZnO NPs among the large particles of adhesives.

Disc Diffusion Test

**Figure 3:**
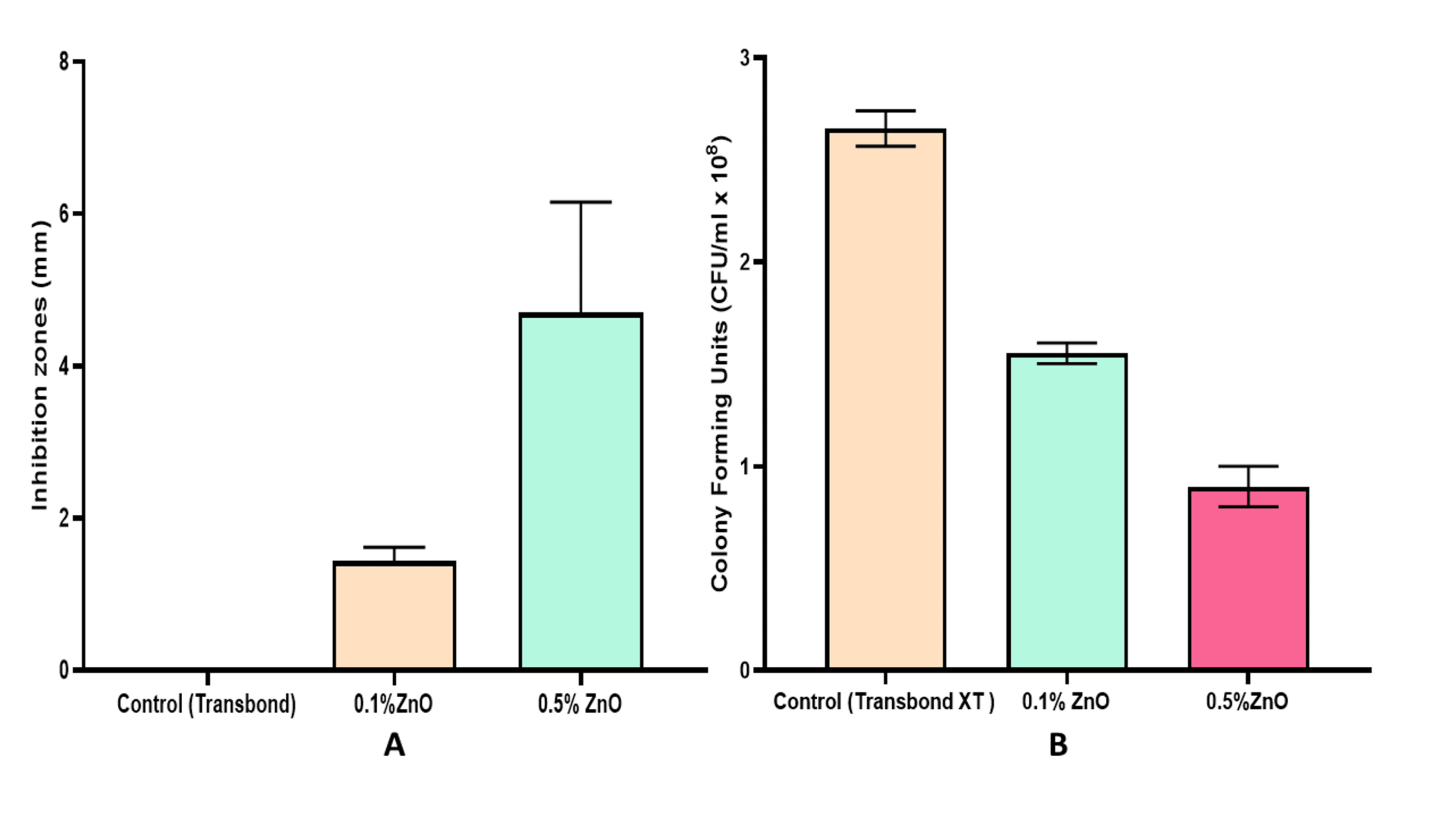
A - Bar graph showing the comparison between mean inhibition zones of different study groups - results of disc diffusion test, B - Bar graph showing the results of the colony-forming test for different study groups

Disc diffusion test results showed that adding 0.1% and 0.5% of ZnO NPs to dental composites results in a zone that inhibits the growth of specific strains of the Streptococcus mutans bacteria. The disc diffusion test showed that the ZnO orthodontic adhesives had antibacterial activity against the test Streptococcus mutans after 48 hours. Additionally, 0.1% and 0.5% ZnO-based orthodontic adhesives produced 1.43 mm and 4.6 mm diameter inhibition zones, respectively. One-way ANOVA statistical analysis found significant differences between the control and experimental groups. Results appear in Figure 3 and Table 2.

**Table 2:** Results of the disc diffusion test and colony-forming test for comparison between groups One-way ANOVA assay was used for comparison between the study groups and p-value was considered significant at p<0.05. ANOVA: analysis of variance

Study Groups	Mean Inhibition Zones (mm)	CFU/ml x 10^8^	Significance (p < 0.05)
Control group (Transbond XT)	0	2.67	0.0001
Experimental group with 0.1% ZnO nanofiller	1.43	1.4	0.0001
Experimental group with 0.5% ZnO nanofiller	4.6	0.9	0.0001

Colony-Forming Test

Experimental groups and control groups differed significantly from one another. With an increase in nanofiller content, CFUs were statistically significantly reduced, with the greatest reduction in CFU/ml observed with adding 0.5% ZnO NPs in orthodontic adhesive. Results of the colony-forming test appear in Table 2 and Figure 3.

Shear Bond Strength Test

**Figure 4:**
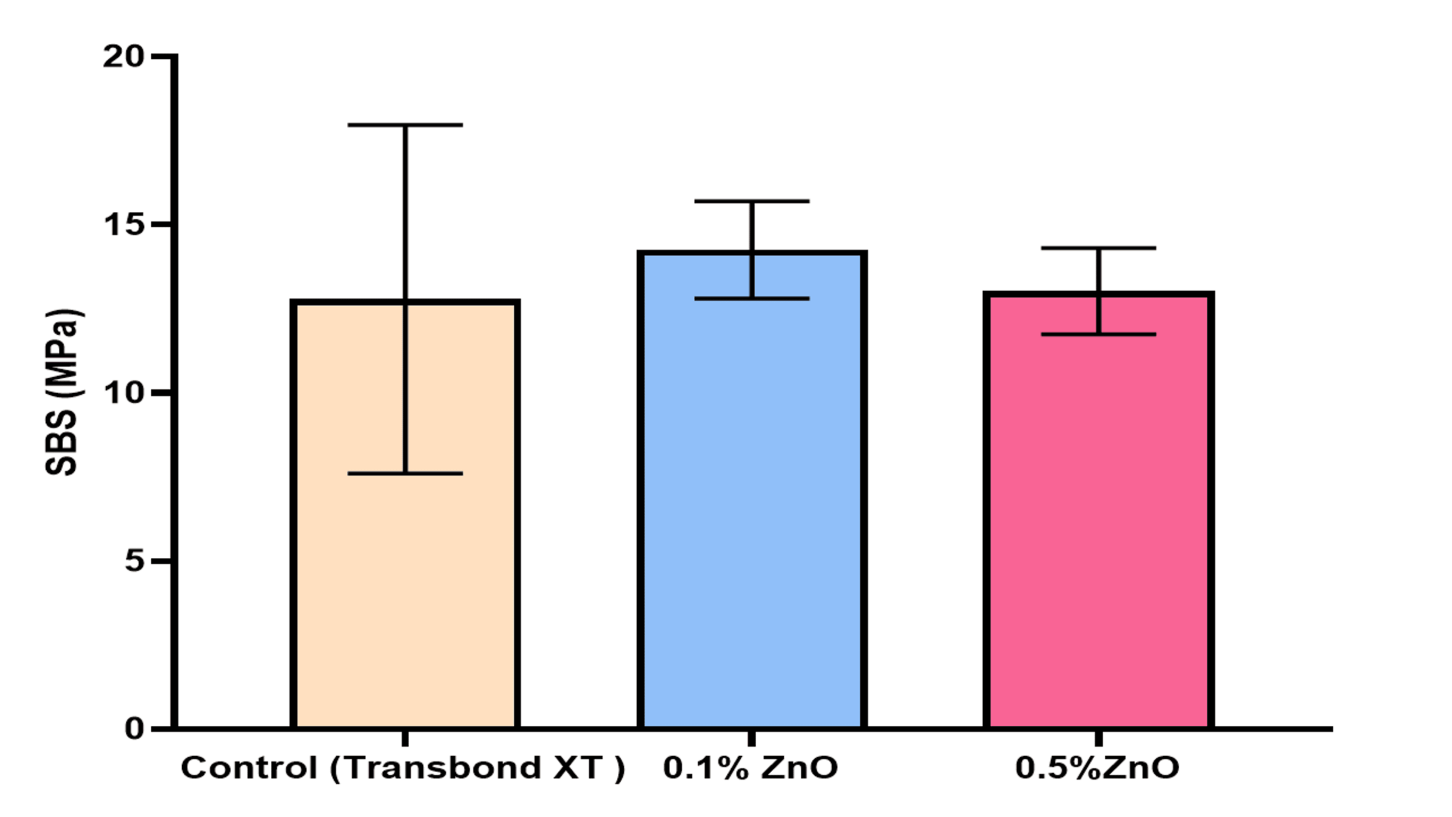
Bar graph showing the comparison of mean shear bond strengths of study groups

**Figure 5:**
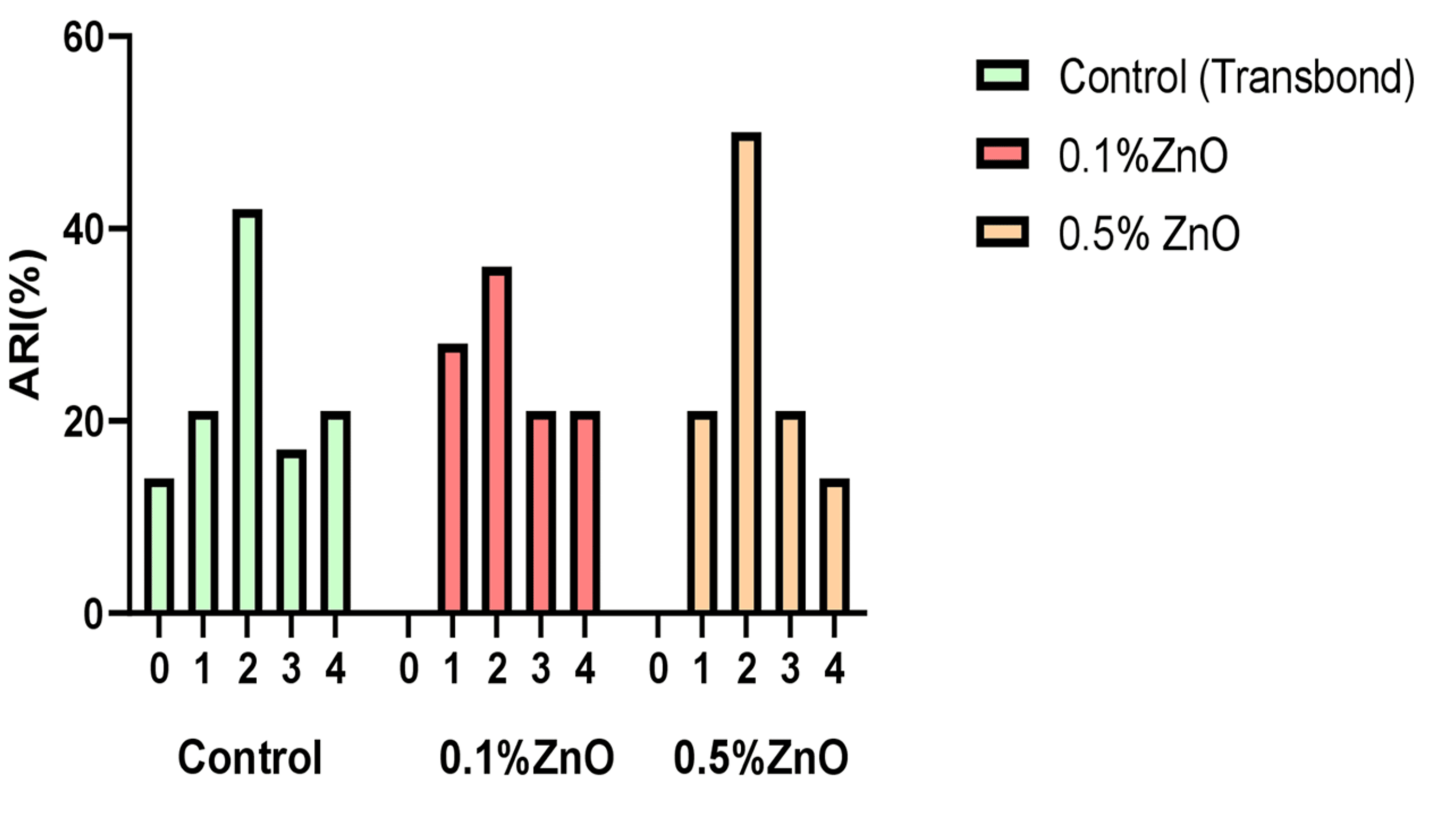
ARI score: comparison of ARI between study groups ARI: adhesive remnants index

The SBS of the experimental adhesive groups -ZnO-based adhesives (0.1% and 0.5% each) - was compared to that of the Transbond XT light cure adhesive (3M Unitek). Table 3 provides descriptive statistics of SBS for research groups. A comparison of SBS value between experimental groups is given by the box and whisker plot in Figure 4. The results of the ARI appear in Figure 5 below. The chi-square test proved statistically nonsignificant, that is, p = 0.29 (p > 0.05).

**Table 3:** Descriptive statistics of shear bond strength testing of study groups: mean shear bond strength The shear bond strength between study groups was found to be statistically nonsignificant (p>0.05).

Study Groups	Minimum	Maximum	Mean ± SD	Statistical Significance (p < 0.05)
Control group (Transbond XT)	9.12	16.45	12.78 ± 5.18	0.76
Experimental group with 0.1% ZnO nanofiller	13.23	15.27	14.25 ± 1.44	0.89
Experimental group with 0.5% ZnO nanofiller	12.12	13.93	13.02 ± 1.28	0.94

## Discussion

It is well-recognized that fixed orthodontic appliances put patients at a greater risk of developing bacterial plaque and demineralizing their enamel. One of the best ways to prevent and minimize enamel demineralization is incorporating different antibacterial agents into orthodontic adhesives that render the area around the bracket resistant to bacterial adherence and biofilm formation [17]. Because of their well-known antibacterial action, biocompatibility, low cytotoxicity, and white color, we have employed ZnO NPs [18].

This study aimed to determine the antibacterial effect of ZnO after incorporation into the orthodontic adhesive in different percentages (0.5% and 0.1%) as nanofillers and their influence on the mechanical characteristics (SBS, ARI) of the orthodontic adhesive. It enabled the determination of filler concentration in the adhesive, offering better bond strength for orthodontic treatment.

The disc diffusion test and colony-forming test were used to determine the experimental orthodontic composite's antibacterial properties. The current study's antibacterial results suggested improvement in the orthodontic bonding adhesive's antibacterial characteristics with the addition of various ZnO concentrations of 0.1% and 0.5%, respectively. Increasing the concentration of NPs demonstrated a steady rise in the suppression of microbial biofilm development, similar to prior studies [18-19].

The results of the antibacterial assay represented by Figure 3 suggested a statistically significant difference in antibacterial properties compared to the conventional Transbond XT (the control). The susceptibility of Streptococcus mutans and the pace at which ZnO NPs diffuse through the medium agar correlate with the diameter of the inhibitory zone in the disc agar diffusion (DAD) test [20]. Compared to ZnO-based orthodontic adhesives, the 0.5% ZnO revealed a more significant inhibition zone than the 0.1% ZnO-based adhesive. The results of the colony-forming test revealed a dose-dependent decrease in the quantity of CFUs with experimental ZnO-based adhesives compared to control ones (Transbond XT).

Mirhashemi et al. investigated the consequences of chitosan and ZnO NP addition on the antibacterial properties of orthodontic bonding adhesives. At varied doses (1%, 5%, and 10%), we investigated the antibacterial influence of these NPs on the growth of Streptococcus mutans, Streptococcus sanguinis, and Lactobacillus acidophilus as both planktonic and biofilm. The results demonstrated that NPs could only significantly enhance the composite's antibacterial activities at a 10% concentration [21]. The outcomes of the current study contrast with those of Mirhashemi et al. in that the present study shows statistically significant antibacterial properties at 0.5%. Increasing the concentration of NPs demonstrated a steady rise in the suppression of microbial biofilm development, similar to prior studies [22].

Generally, stronger bond strengths are preferred in restorative dentistry, whereas ideal bond strengths are needed in orthodontics. A weaker bond strength can frequently cause debonded brackets, whereas a stronger bond can harm the enamel surface of the teeth. According to this study, the control group (Transbond XT) had the highest SBS at 16.45. However, the difference between the control and experimental groups was not statistically significant (p > 0.05). The mean SBS of 0.1% ZnO-based and 0.5% ZnO-based experimental adhesive samples was more significant than Reynolds' recommended minimum clinically acceptable SBS for orthodontic brackets (5-8 MPa). This study's results align with Spencer et al., who determined that SBS decreases as ZnO levels increase in the orthodontic adhesive [23]. They found that the mean SBS for the 13% and 23.1% ZnO nanocomposite was 5.04 MPa and 4.56 MPa, respectively. Another study found that SBS increased when ZnO content was reduced [24].

A comparison of the SBS of the composite after the addition of TiO and ZnO by Jazi et al. [25] revealed results similar to those of our study. In contrast, adding ZnO in a concentration of less than 1% was found to have no statistically significant effect on SBS. However, there was a notable decrease in SBS when ZnO NPs were added in concentrations greater than 5% [24-26].

No statistically significant difference was found between the study groups in our ARI score results (p = 0.29/p > 0.05) calculated by the chi-square test. These results aligned with those of previous studies, which added ZnO, silver/ZnO, ZnO/chitosan, curcumin-doped ZnO, CuO, and TiO2 as nanofillers in orthodontic adhesives [25-29]. Our results are also supported by the systematic review that inferred that the addition of nanofillers ≤ 5 wt% in orthodontic adhesives has no significant effect on bracket-enamel bond strength and ARI score [23].

In our study, the ARI scoring of maximum samples for the control group (Transbond XT) lies in score 2 (42%); 25% to 50% of the composite persisted on the bracket surface, suggesting cohesive failure. In the 0.1% and 0.5% ZnO experimental groups, the maximum distribution of ARI scores was seen in score 2, where 25-50% of the adhesive remains on the tooth surface. Increasing the nanofiller concentration resulted in more adhesive left behind on the bracket surface, resulting in bond failure at the tooth-adhesive interface. However, the differences between the ARI scores of the study groups were nonsignificant.

Limitations and recommendations

Streptococcus mutans was the only type of bacteria tested for antibacterial activity in this study because it is the primary factor in bacterial colonization and biofilm development. Further studies are required to evaluate the antimicrobial activity of these NPs in challenging cariogenic media and to determine the extent (duration) to which this action can be observed. To examine the preventive effects of NPs on the formation of WSLs, future work could use a variety of bacterial species, including S. mutans. Future research is needed to find the ideal therapeutic dose and ensure no long-term adverse effects by examining how cells and tissues may react to NP-based orthodontic adhesives. Despite the inherent drawbacks of an in vitro investigation and its inability to accurately reproduce the intricate oral environment, the results of this study offer valuable information and constitute a crucial first step toward comprehending the bond strength dynamics of orthodontic materials.

## Conclusions

These novel orthodontic adhesives might help prevent WSLs, a challenging problem around the brackets usually seen during and after fixed orthodontic therapy. Compared to conventional orthodontic adhesives (Transbond XT), experimental adhesives containing 0.1% ZnO and 0.5% ZnO as nanofillers showed substantial antibacterial activity. The ZnO addition as nanofiller in concentrations of 0.1% and 0.5% did not yield statistically significant differences in mean SBS values across all groups; the control group displayed the greatest SBS, followed by 0.1% ZnO and 0.5% ZnO. Orthodontic adhesives developed through this research enhance their antibacterial properties by adding cheap, biocompatible, and easily accessible substances (i.e., ZnO). These novel orthodontic adhesives might help prevent WSLs.
